# Test your knowledge and understanding

**Published:** 2019-05-13

**These questions are designed to help you to test your own understanding of the concepts covered in this issue.**

**Figure:**
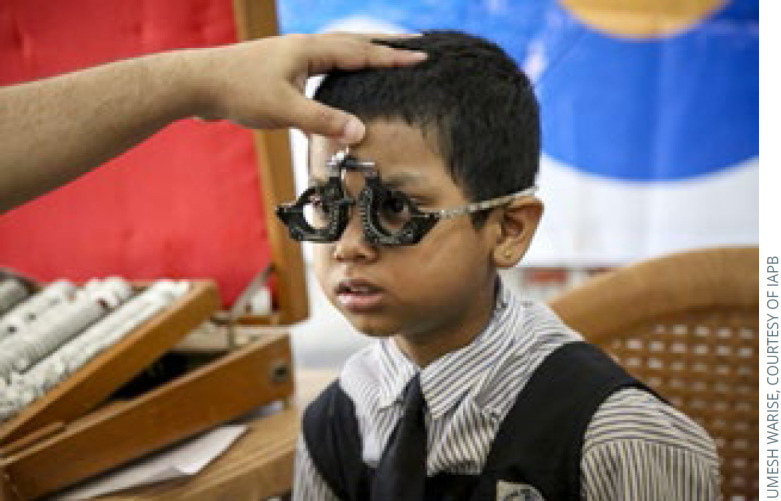
A child is measured for spectacles. INDIA

We hope that you will also discuss the questions with your colleagues and other members of the eye care team, perhaps in a journal club. To complete the activities online – and get instant feedback – please visit **www.cehjournal.org**

**Tick ALL that are TRUE**

**Question 1 When considering the management of myopic children:**
  □ **a.** It does not matter at what age the intervention is implemented; it has the same effect throughout childhood
  □ **b.** Under-correction using spectacles is preferred as the first choice of intervention
  □ **c.** There is not much difference between the amount of myopic progression resulting from the use of progressive addition lenses (PALs) and executive bifocal spectacles in children
  □ **d.** It is possible that combining orthoK with low-dose atropine to reduce the rate of myopia progression has an additive effect
**Question 2 In children at risk of developing myopia:**
  □ **a.** It is advised that they spend a minimum of 90 minutes of outdoor time daily
  □ **b.** It is ideal to compare non-cycloplegic refractions over at least 12 months
  □ **c.** Measuring axial length increase is recommended to predict myopia progression
  □ **d.** It is a good idea to record data on ethnicity, family history of myopia, time spent outdoors and time spent on near work
**Question 3 When detecting myopia**
  □ **a.** It is important to ask questions about the person's family eye health history
  □ **b.** Visual acuity should always be measured for both eyes separately, followed by pinhole acuity
  □ **c.** If pinhole acuity improves, this suggests the patient is myopic. It is not necessary to do any further eye health checks
  □ **d.** Multiple pinholes are easier for young children to use than single pinholes
**Question 4 Considering the myopia epidemic**
  □ **a.** Myopia is currently associated with an increase in urbanisation, reduced educational pressures and moderate near work
  □ **b.** Children are more likely to develop high myopia (≤ −5 D) if they become myopic at a young age (6–8 years old)
  □ **c.** One strategy is for all school children to spend time outdoors as this delay or prevent the onset of myopia, and slow down myopia progression
  □ **d.** A 25% reduction in incidence among primary school children would mean a significant delay in onset of myopia and perhaps high myopia

## ANSWERS

1. a. False. It is essential to implement the intervention during the early years of childhood. b. False. Evidence of the effect of under-correction of myopia is weak and this strategy is not recommended. c. False. PAL spectacles with +2D near addition: 24% control over 3 years. Executive bifocal spectacles with +1.5D near addition: 50% control over 3 years. d. True.
2. a. True. b. False. Cycloplegic refraction is more accurate. c. False. Measuring axial length increase may assist when assessing children prescribed orthokeratology. However, be aware that axial length increases with age, even in children with emmetropia. d. True.
3. a. True. Myopia is heritable, particularly if both parents have myopia. However, it also depends on environmental factors. b. False. Pinhole acuity is only indicated when a patient is not able to see the 6/6 line. c. False. A full eye health check is always required, even if pinhole acuity improves the vision. d. True.
4. a. False. Myopia is currently associated with increased educational pressures and excessive near work, particularly in East Asian populations. b. True. Myopia progresses faster in children who are young (6–8 years old, or even younger). Myopia continues to progress until adolescence, so younger children also have more time during which their myopia can develop. These children are therefore more likely to have high myopia when they enter adulthood. c. False – spending time outdoors does not slow down the progression of myopia in those children that are already myopic. However, if children become myopic only when they are older, their myopia will progress more slowly, as myopia progression is slower in older children (10–12 years old) than in younger children (6–8 years old). d. True.

